# Supplementary material for: Automating region selection with genetic algorithms for energy landscape analyses of brain dynamics
Source: Patterns (N Y). 2026 May 11;7(7):101560. doi: 10.1016/j.patter.2026.101560 (PMC13366526; doi:10.1016/j.patter.2026.101560)
Supplement: Document S1. Figures S1–S11, Tables S1–S5, and supplemental methods [file mmc1.pdf]

Patterns, Volume *7*

Supplemental information

Automating region selection with genetic algorithms for energy landscape analyses of brain dynamics

Koichiro Mori, Tomoyuki Hiroyasu, and Satoru Hiwa

Table S1. Brain regions significantly selected in ELA/GAopt (Scenario 1, on Creativity data; Significant results in bold; FDR-corrected p-values)

				Data validation			
Region	Count	FDR-corrected p	Cohen's h	x	y	z	Network
occipital 142	99	**<1E-10**	2.436	-29	-75	28	occipital
angular gyrus 117	92	**<1E-10**	2.063	51	-59	34	default
post occipital 153	88	**<1E-10**	1.929	29	-81	14	occipital
occipital 139	83	**<1E-10**	1.786	29	-73	29	occipital
dACC 27	40	**<1E-10**	0.864	9	20	34	cingulo-opercular
post occipital 156	38	**<1E-10**	0.823	-37	-83	-2	occipital
mid insula 44	36	**<1E-10**	0.781	37	-2	-3	cingulo-opercular
aPFC 3	35	**<1E-10**	0.761	-29	57	10	fronto-parietal
vFC 40	31	**<1E-10**	0.676	-48	6	1	cingulo-opercular
aPFC 8	29	**<1E-10**	0.632	27	49	26	cingulo-opercular
precentral gyrus 67	28	**<1E-10**	0.610	-54	-22	22	sensorimotor
precuneus 105	28	**<1E-10**	0.610	5	-50	33	default
frontal 32	24	**5.40E-08**	0.519	58	11	14	sensorimotor
inf temporal 91	24	**5.40E-08**	0.519	-61	-41	-2	default
angular gyrus 102	21	**4.55E-06**	0.447	-41	-47	29	cingulo-opercular
temporal 82	18	**0.000230**	0.371	-41	-37	16	sensorimotor
med cerebellum 120	17	**0.000723**	0.345	-6	-60	-15	cerebellum
post occipital 154	13	**0.0428**	0.232	33	-81	-2	occipital

Table S2. Brain regions significantly selected in ELA/GAopt (Scenario 1, on HCP-YA data; Significant results in bold; FDR-corrected p-values)

Region	Count	FDR-corrected p	Cohen's h	Data validation x	y	z	Network
Visual_155	96	**<1E-10**	2.258	-14	-91	31	Visual
Visual_162	92	**<1E-10**	2.087	24	-87	24	Visual
Visual_145	86	**<1E-10**	1.893	8	-72	11	Visual
Visual_149	85	**<1E-10**	1.864	-24	-91	19	Visual
Sensory_Somatomotor_Mouth_44	83	**<1E-10**	1.810	51	-6	32	Sensory
Sensory_Somatomotor_Mouth_42	82	**<1E-10**	1.784	-49	-11	35	Sensory
Visual_170	80	**<1E-10**	1.733	6	-81	6	Visual
Visual_146	75	**<1E-10**	1.613	-8	-81	7	Visual
Sensory_Somatomotor_Mouth_45	73	**<1E-10**	1.567	-53	-10	24	Sensory
Visual_167	72	**<1E-10**	1.545	-3	-81	21	Visual
Visual_156	62	**<1E-10**	1.332	15	-87	37	Visual
Sensory_Somatomotor_Hand_27	51	**<1E-10**	1.109	-38	-27	69	Sensory
Sensory_Somatomotor_Hand_24	43	**<1E-10**	0.949	-40	-19	54	Sensory
Sensory_Somatomotor_Hand_23	40	**<1E-10**	0.888	-23	-30	72	Sensory
Visual_152	37	**<1E-10**	0.826	-18	-68	5	Visual
Sensory_Somatomotor_Hand_46	26	**4.62E-10**	0.589	66	-8	25	Sensory
Visual_159	23	**7.31E-08**	0.519	15	-77	31	Visual
Sensory_Somatomotor_Hand_34	21	**1.61E-06**	0.471	-21	-31	61	Sensory
Visual_163	16	**0.0014**	0.342	6	-72	24	Visual
Visual_157	15	**0.0043**	0.314	29	-77	25	Visual

Table S3. Brain regions significantly selected in ELA/GAopt (Scenario 2; Significant results in bold; FDR-corrected p-values).

Region	Count	FDR-corrected p	Cohen's h	Data validation x	y	z	Network
Sensory_Somatomotor_Hand_24	98	**<1E-10**	2.410	-40	-19	54	Sensory
Sensory_Somatomotor_Hand_27	98	**<1E-10**	2.410	-38	-27	69	Sensory
Sensory_Somatomotor_Hand_37	97	**<1E-10**	2,346	-38	-15	69	Sensory
Uncertain_142	93	**<1E-10**	2.159	-12	-95	13	Uncertain
Uncertain_2	92	**<1E-10**	2.121	27	-97	-13	Uncertain
Sensory_Somatomotor_Hand_19	88	**<1E-10**	1.987	13	-33	75	Sensory
Sensory_Somatomotor_Hand_18	85	**<1E-10**	1.899	-7	-33	72	Sensory
Sensory_Somatomotor_Hand_34	82	**<1E-10**	1.818	-21	-31	61	Sensory
Sensory_Somatomotor_Hand_28	78	**<1E-10**	1.718	20	-29	60	Sensory
Sensory_Somatomotor_Hand_21	68	**<1E-10**	1.492	29	-17	71	Sensory
Sensory_Somatomotor_Hand_36	66	**<1E-10**	1.449	42	-20	55	Sensory
Sensory_Somatomotor_Hand_23	62	**<1E-10**	1.366	-23	-30	72	Sensory
Sensory_Somatomotor_Hand_31	49	**<1E-10**	1.103	10	-17	74	Sensory
Sensory_Somatomotor_Hand_35	31	**<1E-10**	0.733	-13	-17	75	Sensory

Table S4. Brain regions significantly selected in ELA/GAopt (Scenario 3; Significant results in bold; FDR-corrected p-values).

Region	Count	FDR-corrected p	Cohen's h	Data validation			Network
				x	y	z	
Visual_145	100	**<1E-10**	2.712	8	-72	11	Visual
Visual_146	99	**<1E-10**	2.512	-8	-81	7	Visual
Visual_170	99	**<1E-10**	2.512	6	-81	21	Visual
Visual_167	85	**<1E-10**	1.916	-3	-81	21	Visual
Visual_152	77	**<1E-10**	1.712	-18	-68	5	Visual
Visual_163	73	**<1E-10**	1.619	6	-72	24	Visual
Visual_151	64	**<1E-10**	1.425	-15	-72	-8	Visual
Visual_148	54	**<1E-10**	1.221	20	-66	2	Visual
Visual_156	51	**<1E-10**	1.161	15	-87	37	Visual
Visual_155	45	**<1E-10**	1.041	-14	-91	31	Visual
Visual_162	43	**<1E-10**	1.001	24	-87	24	Visual
Visual_149	34	**<1E-10**	0.815	-24	-91	19	Visual
Visual_159	21	**3.92E-08**	0.522	15	-77	31	Visual
Visual_160	15	**0.000428**	0.366	-16	-52	-1	Visual
DefaultMode_86	12	**0.0168**	0.278	-44	-65	35	Default
Visual_150	11	**0.0463**	0.246	27	-59	-9	Visual

Table S5. Brain regions significantly selected in ELA/GAopt (Scenario 4; Significant results in bold; FDR-corrected p-values).

Region	Count	FDR-corrected p	Cohen's h	Data validation x	y	z	Network
Visual_167	99	**<1E-10**	2.512	-3	-81	21	Visual
Visual_170	98	**<1E-10**	2.428	6	-81	6	Visual
Visual_145	96	**<1E-10**	2.309	8	-72	11	Visual
Visual_146	96	**<1E-10**	2.309	-8	-81	7	Visual
Visual_149	76	**<1E-10**	1.688	-24	-91	19	Visual
Visual_155	73	**<1E-10**	1.619	-14	-91	31	Visual
Visual_163	61	**<1E-10**	1.363	6	-72	24	Visual
Visual_152	58	**<1E-10**	1.302	-18	-68	5	Visual
Visual_156	57	**<1E-10**	1.282	15	-87	37	Visual
Visual_148	36	**<1E-10**	0.857	20	-66	2	Visual
Visual_171	32	**<1E-10**	0.773	-26	-90	3	Visual
Visual_151	23	**1.13E-09**	0.571	-15	-72	-8	Visual
Visual_160	23	**1.13E-09**	0.571	-16	-52	-1	Visual
Visual_162	22	**6.55E-09**	0.547	24	-87	24	Visual
Visual_159	17	**2.42E-05**	0.420	15	-77	31	Visual
DefaultMode_121	14	**0.00141**	0.337	13	30	59	Default
Visual_172	14	**0.00141**	0.337	-33	-79	-13	Visual
Visual_147	13	**0.00416**	0.308	-28	-79	19	Visual
DefaultMode_99	13	**0.00416**	0.308	-16	29	53	Default
Visual_150	13	**0.00416**	0.308	27	-59	-9	Visual
Visual_169	11	**0.0358**	0.246	37	-84	13	Visual
DefaultMode_86	11	**0.0358**	0.246	-44	-65	35	Default

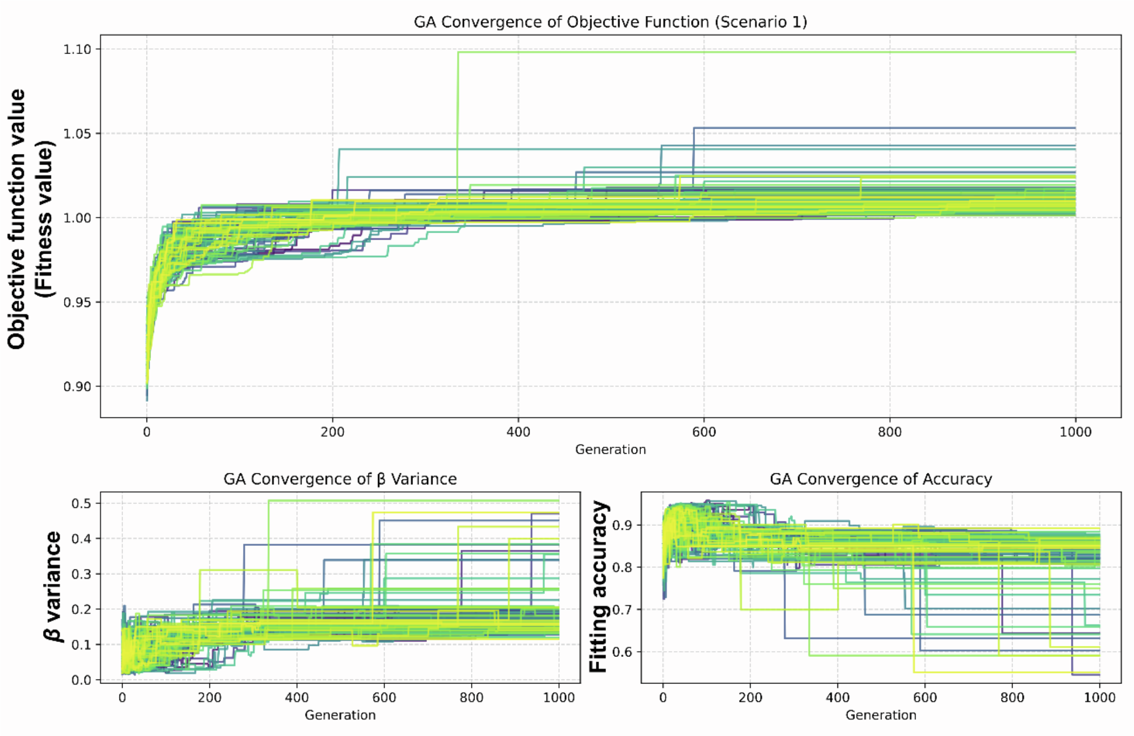

Figure S1. Convergence of the objective function with ELA/GAopt over 1000 generations for 100 runs with different starting population (Scenario 1, on Creativity data). The objective function (top figure) is the sum of the β variance (bottom left) and the pMEM fitting accuracy (bottom right).

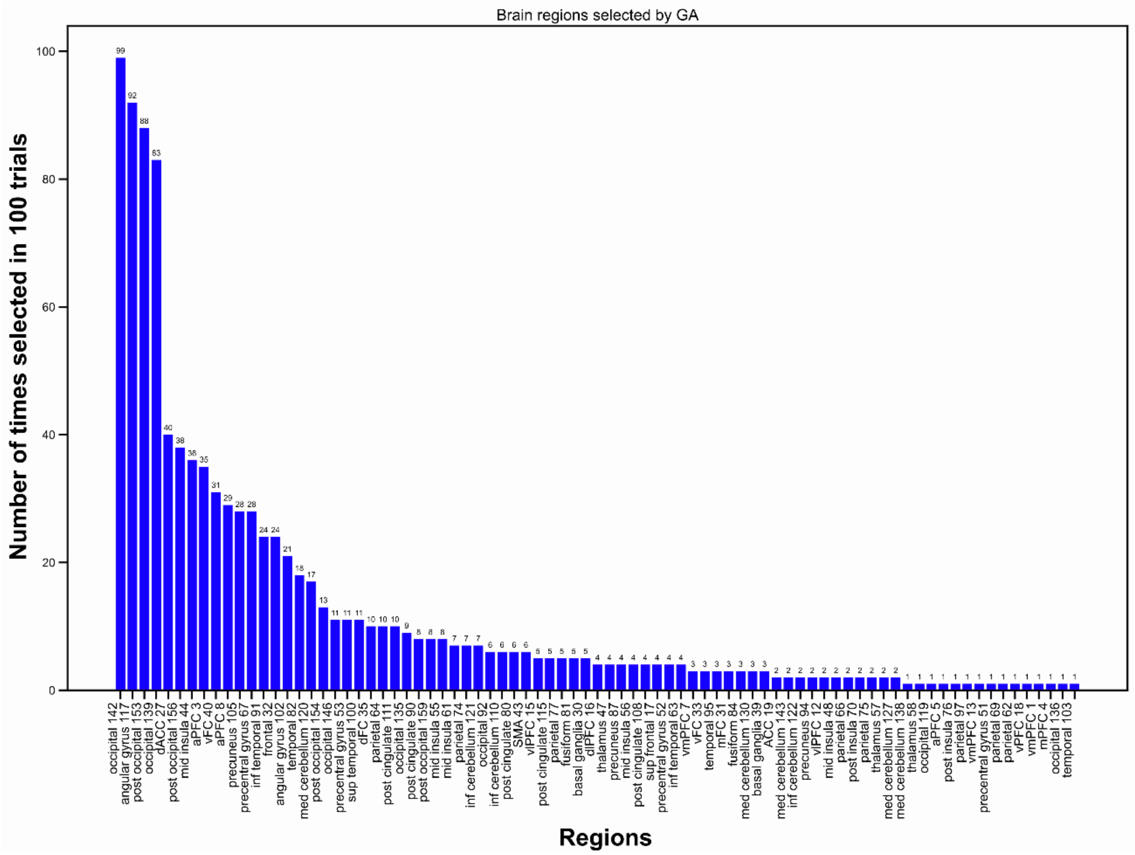

Figure S2. Selection frequency of each ROI across 100 runs of ELA/GAopt (Scenario 1, Creativity data).

A

Number	ROI label	Number	ROI label
0	med cerebellum 120	5	occipital 142
1	aPFC 8	6	occipital 139
2	vFC 40	7	post occipital 156
3	angular gyrus 117	8	post occipital 153
4	inf temporal 91	9	frontal 32

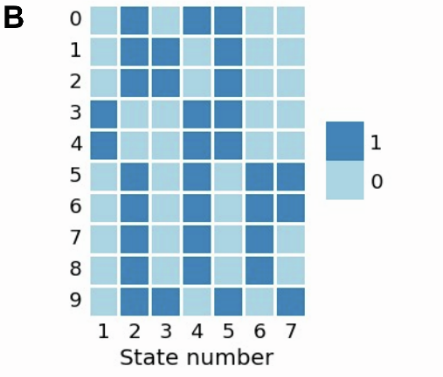

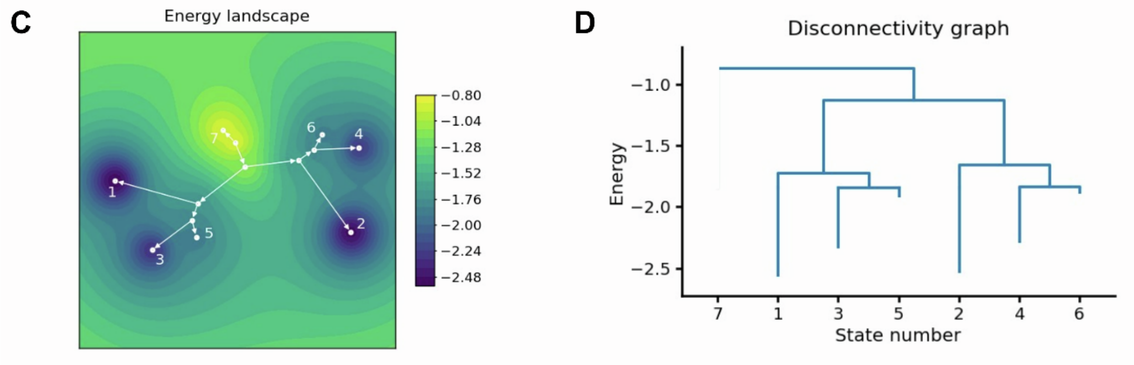

Figure S3. Example of energy landscape analysis using the optimized ROI set specified by a representative run consisting solely of the 18 significantly selected ROIs. (A) List of the selected ROIs. (B) Observed local minimum states. (C) The resulting energy landscape. (D) Disconnectivity graph of the energy landscape.

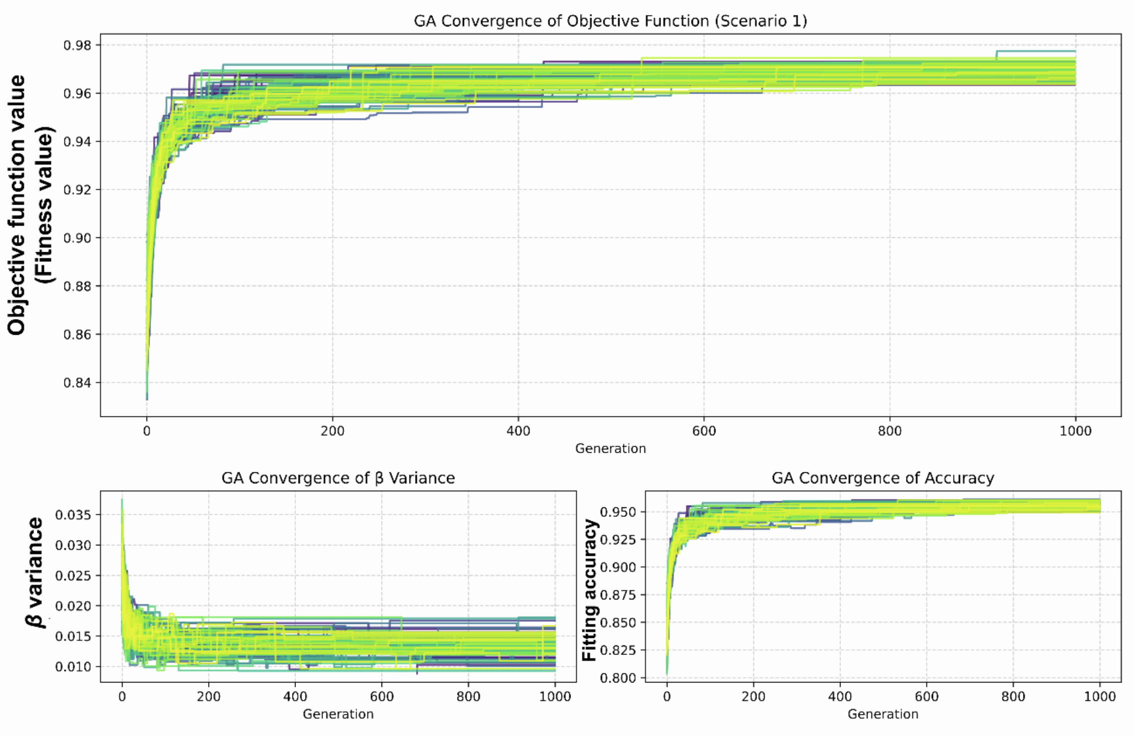

Figure S4. Convergence of the objective function with ELA/GAopt over 1000 generations for 100 runs with different starting population (Scenario 1, on HCP-YA data). The objective function (top figure) is the sum of the β variance (bottom left) and the pMEM fitting accuracy (bottom right).

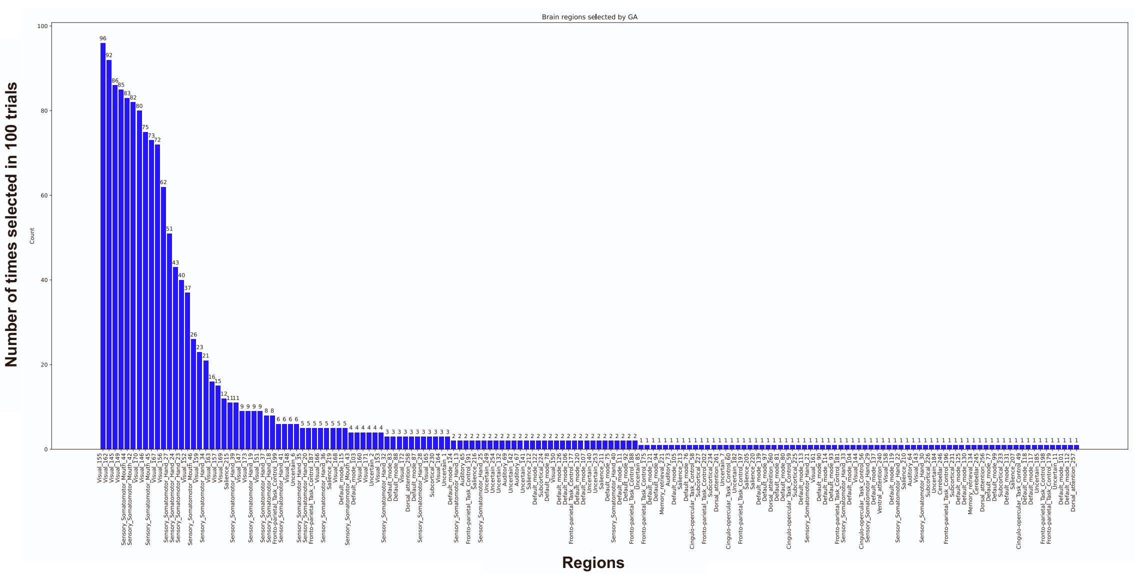

Figure S5. Selection frequency of each ROI across 100 runs of ELA/GAopt (Scenario 1, on HCP-YA data).

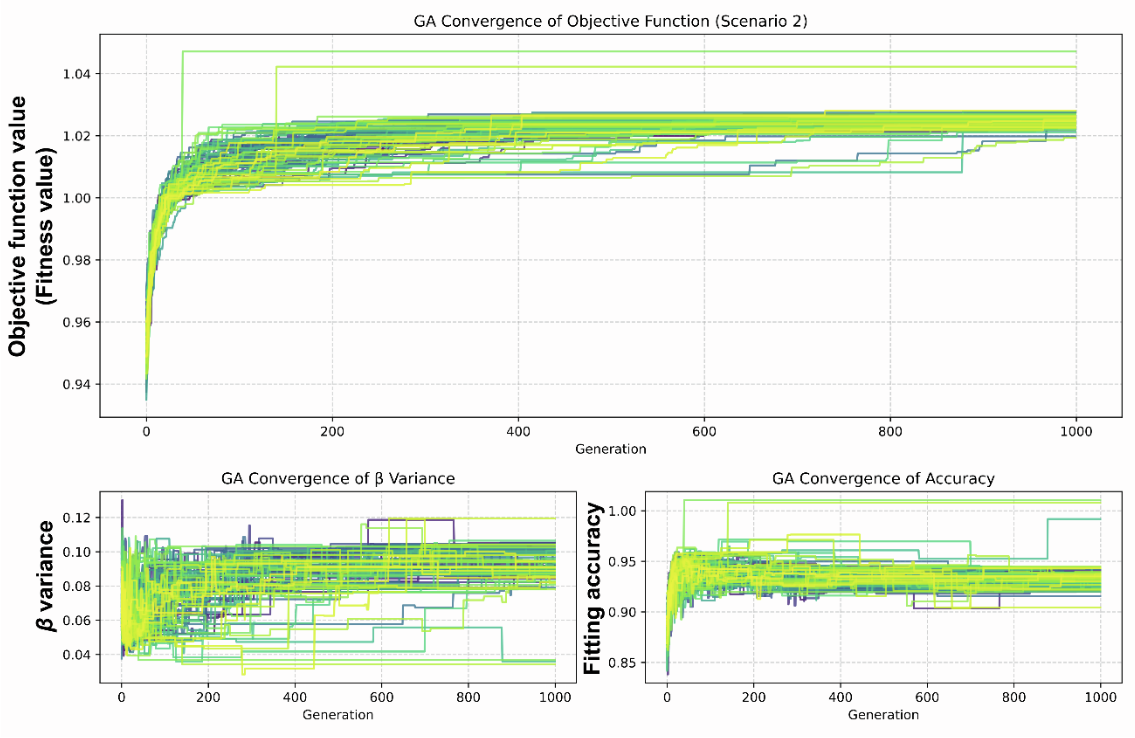

Figure S6. Convergence of the objective function with ELA/GAopt over 1000 generations for 100 runs with different starting population (Scenario 2). The objective function (top figure) is the sum of the β variance (bottom left) and the pMEM fitting accuracy (bottom right).

ELA with Genetic ROI Optimization

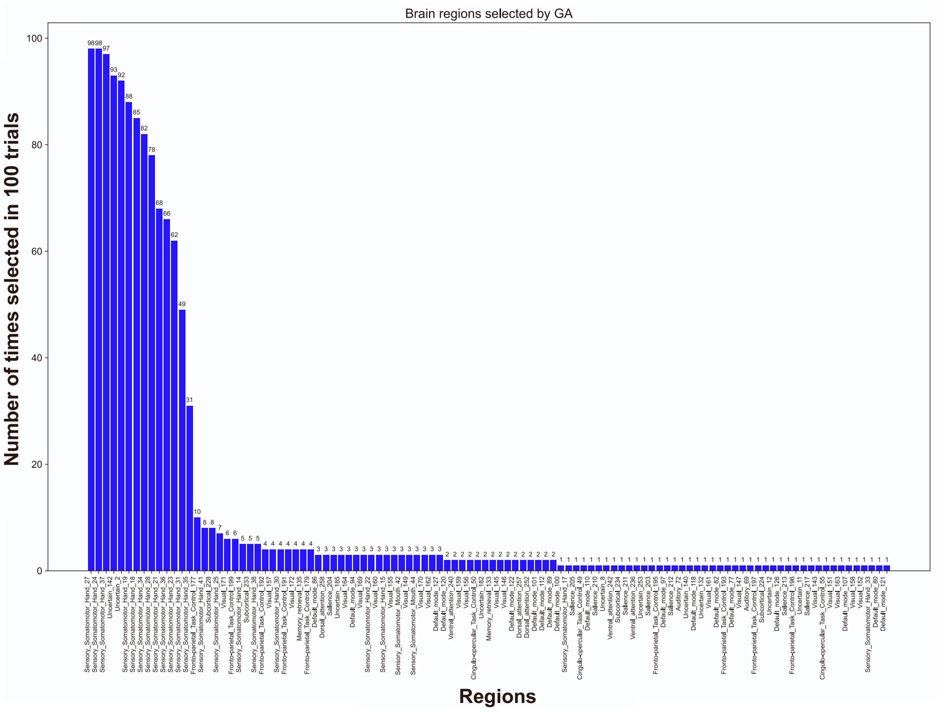

Figure S7. Selection frequency of each ROI across 100 runs of ELA/GAopt (Scenario 2).

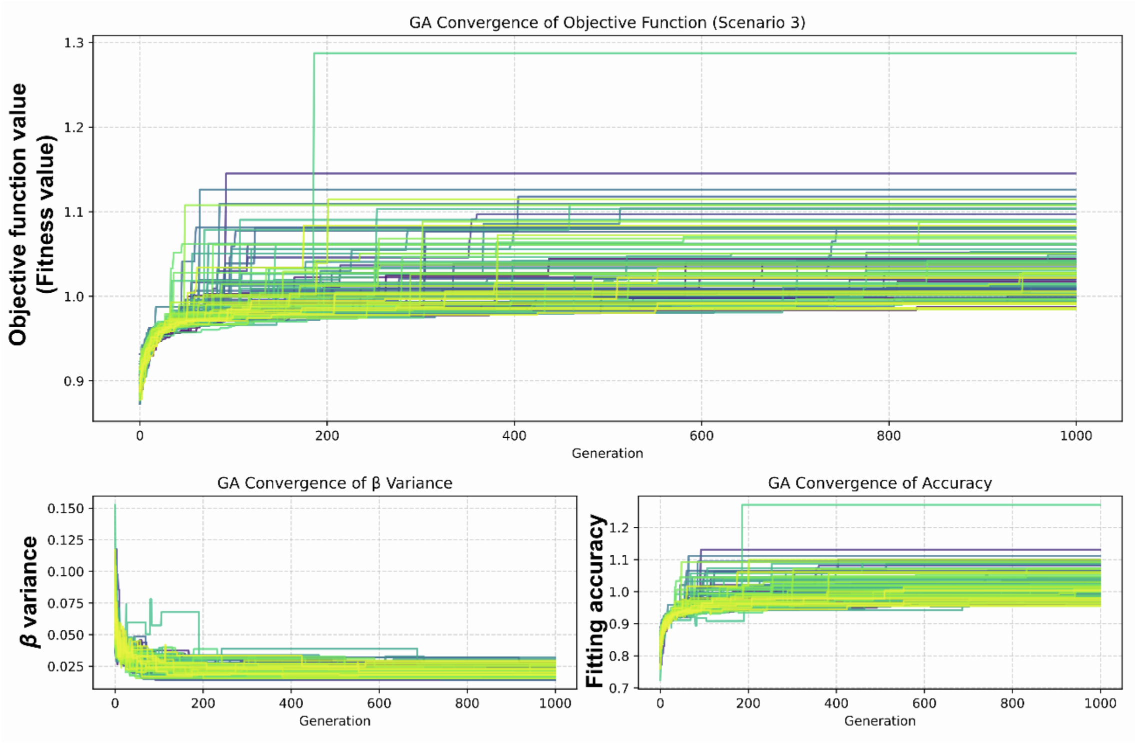

Figure S8. Convergence of the evaluation function across generations during 100 runs of ELA/GAopt (Scenario 3). The objective function (top figure) is the sum of the β variance (bottom left) and the pMEM fitting accuracy (bottom right).

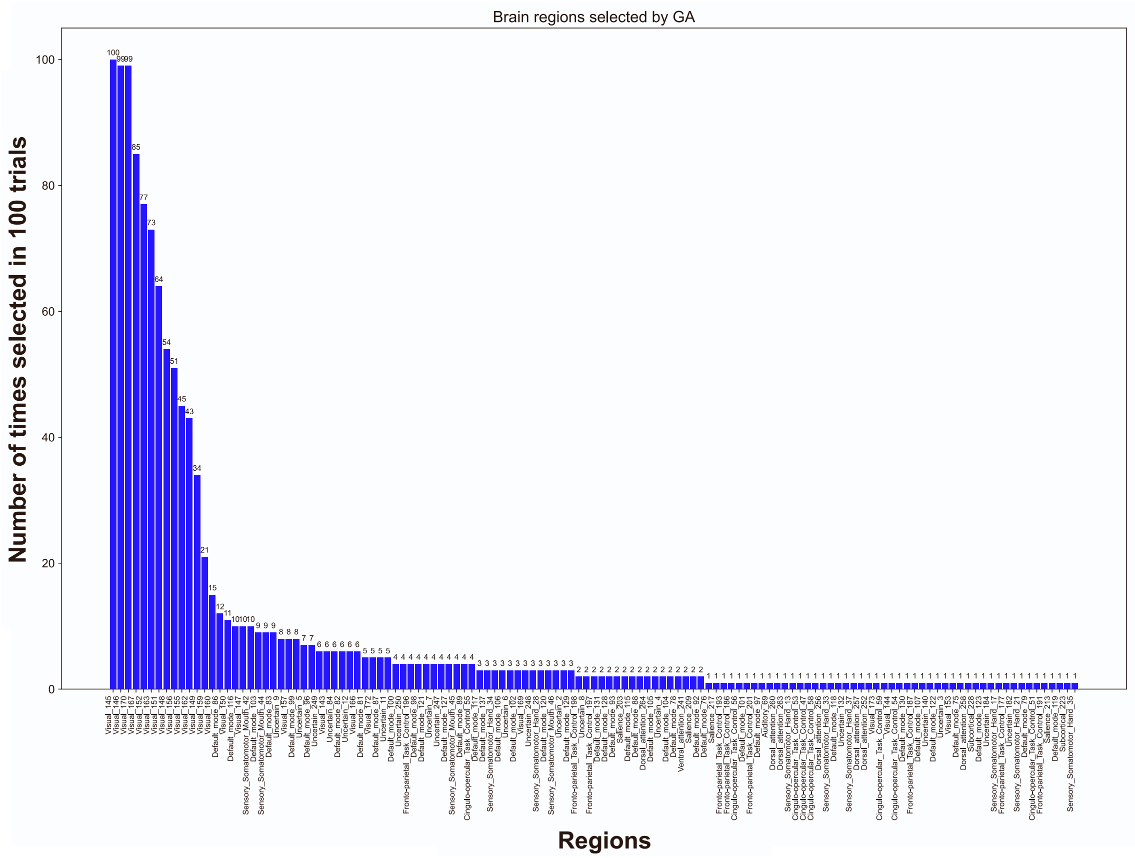

Figure S9. Selection frequency of each ROI across 100 runs of ELA/GAopt (Scenario 3).

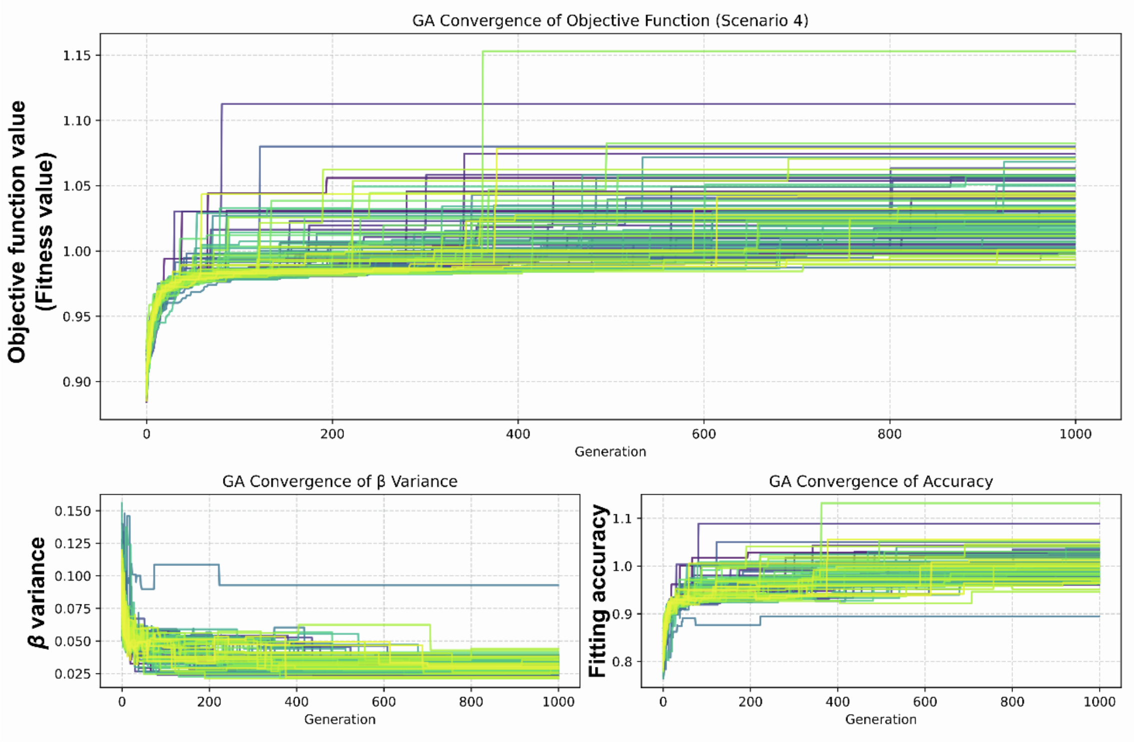

Figure S10. Convergence of the evaluation function across generations during 100 runs of ELA/GAopt (Scenario 4). The objective function (top figure) is the sum of the β variance (bottom left) and the pMEM fitting accuracy (bottom right).

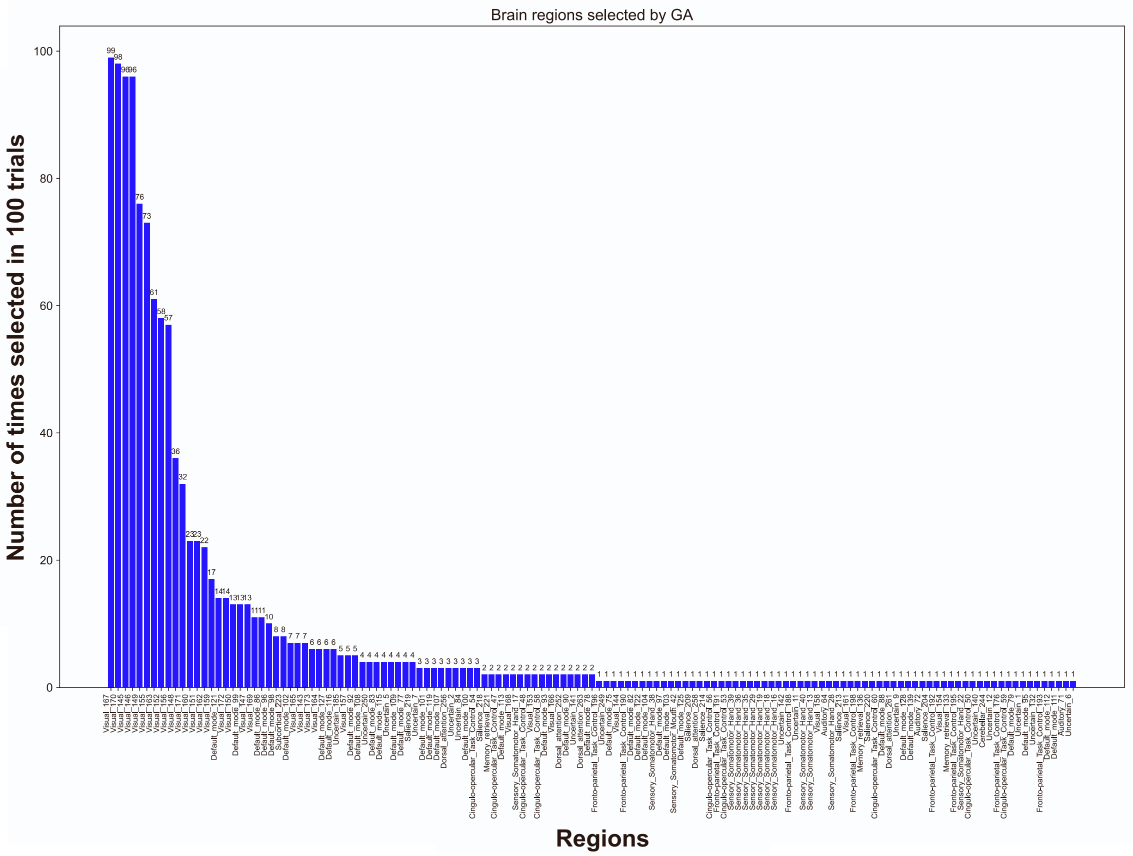

Figure S11. Selection frequency of each ROI across 100 runs of ELA/GAopt (Scenario 4).

SUPPLEMENTARY METHODS

Personalization of pMEM via temperature optimization

In standard ELA, individual data is concatenated into a group-level dataset to estimate a single set of $\boldsymbol{h}$ and $\boldsymbol{J}$ parameters representing group characteristics. Although it is theoretically possible to fit pMEM to individual data, this approach is rarely adopted in practice because it requires a large amount of data to achieve stable estimation. For example, in typical fMRI datasets, the number of time points per individual is often insufficient to reliably estimate all parameters of the model. Therefore, pMEM is typically estimated using concatenated group-level data for robustness and accuracy. However, when individual variability needs consideration, personalization of the pMEM becomes essential. Ruffini et al. proposed a two-step pMEM parameter optimization method in which the inverse temperature parameter β, which characterizes the Boltzmann distribution, is optimized for each individual data while keeping the group-derived $\boldsymbol{h}$ and $\boldsymbol{J}$ parameters fixed. This approach allows group-level trends to be captured by $\boldsymbol{h}$ and $\boldsymbol{J}$, while individual differences are partly parameterized by β within this personalization scheme. Here, we use β as a practical personalization parameter without asserting it as the sole source of inter-individual variability.

Here, the distribution for the individual is defined as:

$$P(\boldsymbol{\sigma}|\boldsymbol{h},\boldsymbol{J},\beta) = \frac{e^{-\beta E(\boldsymbol{\sigma}|\boldsymbol{h},\boldsymbol{J})}}{\sum_{\{\sigma'\}} e^{-\beta E(\boldsymbol{\sigma}'|\boldsymbol{h},\boldsymbol{J})}}$$

The optimization of β is performed by maximizing the approximate log-likelihood using a gradient ascent algorithm. The update rule is:

$$\beta^{\text{new}} - \beta^{\text{old}} = -\varepsilon\left(\langle E\rangle_{\text{empirical}} - \langle E\rangle_{\tilde{P}}\right)$$

Where ε is the step size of the gradient ascent, $\langle E\rangle_{\text{empirical}}$ is the average energy computed from the data, and $\langle E\rangle_{\tilde{P}}$ is the model-based expected energy. These are derived from the log-likelihood expression:

$$\log\mathcal{L}(\boldsymbol{h},J) = \sum_{t=1}^{t_{\max}} \log P(\sigma(t)|\boldsymbol{h},J) = \sum_{t=1}^{t_{\max}} \left(-\beta E(\sigma(t)|\boldsymbol{h},J) - \log Z\left(\boldsymbol{h},J,\sigma_{/\boldsymbol{i}}(t),\beta\right)\right)$$

$$Z(\boldsymbol{h},J,\beta,t) = \sum_{\sigma_i'} \exp\left[-\beta E_i(\sigma_i'|\boldsymbol{h},J,\sigma_{/i}(t))\right]$$

$$\langle E\rangle_{\tilde{P}} = -h_i\langle\sigma_i\rangle_{\tilde{P}} - \sum_{j\neq i\leq N} J_{ij}\langle\sigma_i\sigma_j\rangle_{\tilde{P}}$$

This procedure enables the personalization of the energy landscape while preserving group-level structure. The implementation in this study closely followed the approach described by Ruffini et al. and all equations and optimization steps are reproduced here for clarity and reproducibility.

Data preprocessing

Three independent datasets, the Creativity dataset, the ABIDE II dataset and HCP-YA dataset, were preprocessed in the following steps. It should be noted that the ABIDE II and HCP-YA data were originally provided in non-BIDS format and were converted to BIDS format using the procedure outlined by Ran et al. (https://github.com/thebrisklab/ABIDE_Preprocessing) [S1] and Suyash et al (https://github.com/suyashdb/hcp2bids). [S2] For the ABIDE II dataset, the first five volumes were excluded to remove non-equilibrium effects of magnetization. This exclusion step was not applied to the Creativity dataset due to its limited number of subjects and time points. For the HCP-YA dataset, no slice timing correction was performed following to the prior study. [S3] Functional MR images from both datasets were then preprocessed using fMRIPrep version 23.1.4, a robust and standardized preprocessing pipeline built on Nipype.

First, a reference volume was generated from each run to use as a reference for head motion correction. The head motion parameters (transformation matrix and six parameters corresponding to translations and rotations) for this reference image were then estimated before spatiotemporal filtering was performed. Slice timing correction was further performed to correct the temporal misalignment of each slice. The EPI image was then aligned with the T1w image in six degrees of freedom using boundary-based registration.

Several confounding time series were subsequently estimated from the preprocessed BOLD data, including framewise displacements (FD), DVARS, and global signals from the cerebrospinal fluid (CSF), white matter (WM), and whole-brain mask.

Further preprocessing was carried out using the Nilearn package in Python. [S4] This included spatial smoothing with a Gaussian kernel (FWHM = 6 mm), high-pass filtering at 0.01 Hz, low-pass filtering at 0.1 Hz, and regressing out of confounding variables ("high-pass", "motion", "wmcsf", "scrub"). The denoising strategy followed established procedures in prior studies. [S5]

Additionally, we used different atlases for each dataset: the Dosenbach atlas for Creativity Dataset ($d = 160$), [S6–10] and the Power 264 atlas for HCP-YA and ABIDE II Dataset ($d = 264$), [S11] respectively. The BOLD time courses were extracted from the spherical mask with a 4 mm radius centered at each ROI coordinate, then averaged within the ROI. The average time courses were considered representative of brain activity in each ROI. Finally, the ROI-averaged activity data was then binarized using the threshold of the time-averaged value of each activity for the ELA input.

References

S1. thebrisklab/ABIDE_Preprocessing (2024). (Brain Research in Imaging Statistics Kit).

S2. Suyash (2025). suyashdb/hcp2bids.

S3. Smith, S.M., Beckmann, C.F., Andersson, J., Auerbach, E.J., Bijsterbosch, J., Douaud, G., Duff, E., Feinberg, D.A., Griffanti, L., Harms, M.P., et al. (2013). Resting-state fMRI in the Human Connectome Project. NeuroImage *80*, 144–168. https://doi.org/10.1016/j.neuroimage.2013.05.039.

S4. Nilearn Nilearn. https://nilearn.github.io/index.html.

S5. Wang, H.-T., Meisler, S.L., Sharmarke, H., Clarke, N., Gensollen, N., Markiewicz, C.J., Paugam, F., Thirion, B., and Bellec, P. (2023). Continuous Evaluation of Denoising Strategies in Resting-State fMRI Connectivity Using fMRIPrep and Nilearn. BioRxiv Prepr. Serv. Biol., 2023.04.18.537240. https://doi.org/10.1101/2023.04.18.537240.

S6. Dosenbach, N.U.F., Nardos, B., Cohen, A.L., Fair, D.A., Power, J.D., Church, J.A., Nelson, S.M., Wig, G.S., Vogel, A.C., Lessov-Schlaggar, C.N., et al. (2010). Prediction of Individual Brain Maturity Using fMRI. Science *329*, 1358–1361. https://doi.org/10.1126/science.1194144.

S7. Dosenbach, N.U.F., Visscher, K.M., Palmer, E.D., Miezin, F.M., Wenger, K.K., Kang, H.C., Burgund, E.D., Grimes, A.L., Schlaggar, B.L., and Petersen, S.E. (2006). A Core System for the Implementation of Task Sets. Neuron *50*, 799–812. https://doi.org/10.1016/j.neuron.2006.04.031.

S8. Dosenbach, N.U.F., Fair, D.A., Miezin, F.M., Cohen, A.L., Wenger, K.K., Dosenbach, R.A.T., Fox, M.D., Snyder, A.Z., Vincent, J.L., Raichle, M.E., et al. (2007). Distinct brain networks for adaptive and stable task control in humans. Proc. Natl. Acad. Sci. *104*, 11073–11078. https://doi.org/10.1073/pnas.0704320104.

S9. Fair, D.A., Cohen, A.L., Power, J.D., Dosenbach, N.U.F., Church, J.A., Miezin, F.M., Schlaggar, B.L., and Petersen, S.E. (2009). Functional Brain Networks Develop from a “Local to Distributed” Organization. PLOS Comput. Biol. *5*, e1000381. https://doi.org/10.1371/journal.pcbi.1000381.

S10. Fox, M.D., Snyder, A.Z., Vincent, J.L., Corbetta, M., Van Essen, D.C., and Raichle, M.E. (2005). The human brain is intrinsically organized into dynamic, anticorrelated functional

networks. Proc. Natl. Acad. Sci. U. S. A. *102*, 9673–9678. https://doi.org/10.1073/pnas.0504136102.

S11. Power, J.D., Cohen, A.L., Nelson, S.M., Wig, G.S., Barnes, K.A., Church, J.A., Vogel, A.C., Laumann, T.O., Miezin, F.M., Schlaggar, B.L., et al. (2011). Functional network organization of the human brain. Neuron *72*, 665–678. https://doi.org/10.1016/j.neuron.2011.09.006.
